# Association between smoking and obstructive sleep apnea based on the STOP-Bang index

**DOI:** 10.1038/s41598-023-34956-5

**Published:** 2023-06-05

**Authors:** Yun Seo Jang, Nataliya Nerobkova, Kyungduk Hurh, Eun-Cheol Park, Jaeyong Shin

**Affiliations:** 1 Department of Public Health, Graduate School, Yonsei University, Seoul, Republic of Korea; 2 Institute of Health Services Research, Yonsei University, 50 Yonsei-to, Seodaemun-Gu, Seoul, 03722 Republic of Korea; 3 Department of Preventive Medicine, Yonsei University College of Medicine, Seoul, Republic of Korea; 4 Department of Policy Analysis and Management, College of Human Ecology, Cornell University, Ithaca, NY USA

**Keywords:** Diseases, Health care, Risk factors, Signs and symptoms

## Abstract

Smoking is a risk factor for respiratory diseases, and it worsens sleep quality due to nicotine stimulation and sudden nicotine withdrawal during sleep. This can increase the severity of OSA through alterations upper airway inflammation and neuromuscular function, arousal mechanisms, and sleep architecture. Therefore, it may lead to sleep-disrupted breathing, particularly obstructive sleep apnea (OSA). Herein, this study aims to research the association between smoking and OSA through the STOP-Bang index. In this study, total sample of 3442 participants (1465 men and 1977 women) were analyzed. We used data from the Korea National Health and Nutrition Examination Survey in 2020 by classifying adults into current, ex-, and non-smokers. A multiple logistic regression analysis was used to investigate the association between smoking and OSA. Furthermore, multinomial regression analysis was used to investigate the effect of smoking cessation. For males, compared to the non-smokers, the odds ratios (OR) for the OSA were significantly higher in the ex-smokers (OR: 1.53, 95% confidence interval(CI) 1.01–2.32) and current smokers (OR: 1.79, 95% CI 1.10–2.89). In females, higher ORs were observed for OSA risk, similar to the non-smokers, smoking cessation, and pack-years. Among men, OSA was significantly associated with a moderate risk for ex-smokers (OR: 1.61, 95% CI 1.05–2.48) and a severe risk for current smokers (OR: 1.88, 95% CI 1.07–3.29). This study observed that smoking might contribute to OSA risk among adults. Smoking cessation can help to manage sleep quality properly.

## Introduction

Obstructive sleep apnea (OSA) is characterized by the repeated cessation of breathing during sleep due to obstruction or collapse of the upper airway. When this symptom occurs, the oxygen saturation of the blood decreases, and sleep is interrupted to breathe. This is also known as the “apnea event.” OSA occurs in 14% of men and 5% of women in the general adult population^1^. The prevalence of OSA increases with obesity^1^, and the five-year incidence in middle-aged adults is 7–11%^2^. Nevertheless, only 1 in 50 patients with symptoms suggesting OSA syndrome is diagnosed and treated^3^. OSA is a common comorbidity in patients with lung diseases, and recognizing its prevalence and clinical significance may improve patients’ quality of life^4^. Particularly, the appropriate and timely circadian rhythm treatment of OSA leads to increased positive outcomes for patients with sepsis, chronic obstructive pulmonary disease, and cancer^5^. The best standard to diagnose OSA is polysomnography. However, it is challenging for use in epidemiological studies because it requires a lot of money and time. Therefore, there was a lot of interest in OSA’s screening tools around the world. OSA’s screening tools include the Berlin questionnaire^6^ and STOP-Bang^7^.

The risks of respiratory diseases in smokers are known worldwide^8,9^. A study that investigated the health outcomes of ex-smokers observed that the risk of lung cancer was reduced^10^. Other studies revealed that smoking cessation for a prolonged duration was associated with a decreased tendency to have cancer and obstructive spirometry pattern^11^. These respiratory diseases are associated with sleep apnea, and there is an association between smoking and sleep disorders, including sleep patterns, sleep maintenance, and daytime sleepiness^12^.

According to a cohort study, smokers have reduced sleep quality, less restorative sleep, and take longer to fall asleep than non-smokers^13–15^. Sleep disorders can affect well-being and mood during waking hours and frequently occur among smokers. A study has detected a decrease in sleep quality and changes in sleep patterns in young, healthy smokers^16^. Some of these changes are based on daily tobacco consumption and serum nicotine levels, and this supports the effect of smoking on sleep patterns^16^.

Previous OSA studies have observed associations with asthma, metabolic syndrome, insulin resistance, hypothyroidism, and depression^17–22^. Furthermore, based on previous studies, a biological association between obstructive sleep apnea and smoking can be found. Smoking is widely known to negatively affect respiratory function, causing inflammation and swelling of the upper airway^23^. This phenomenon can also lead to obstruction and difficulty breathing during sleep, resulting in symptoms such as snoring, panting, and interrupted sleep. We also assume that smoking reduces muscle tension in people and causes structural changes in the upper airway by accumulating pharyngeal fat, which can contribute to the severity of OSA symptoms^24^. Finally, it increases oxidative stress and inflammation throughout the body, leading to sleep disorders and the development of an arousal threshold that makes it easier to wake up^25^.

There is biological plausibility of disorders between smoking and OSA; however, a significant relationship has not been conclusively established within more definitive physiologic studies^26^. Therefore, this study aimed to investigate the association between smoking and OSA using the STOP-Bang index in a representative population of Korean adults^27^.

## Methods

### Data

Data used in this study were obtained from the Korea National Health and Nutrition Examination Survey^28^, a cross-sectional survey. This survey is conducted annually by the Korea Disease Control and Prevention Agency (KDCA). It uses a stratified, multi-stage cluster sampling design based on age, gender, and geographic area^28^. The KNAHNES is reliable for Korean health policies and programs, and it is publicly accessible data. This study did not require ethical approval as the KNHANES complies with the Helsinki Declaration^29^.

### Study population

**Figure 1:**
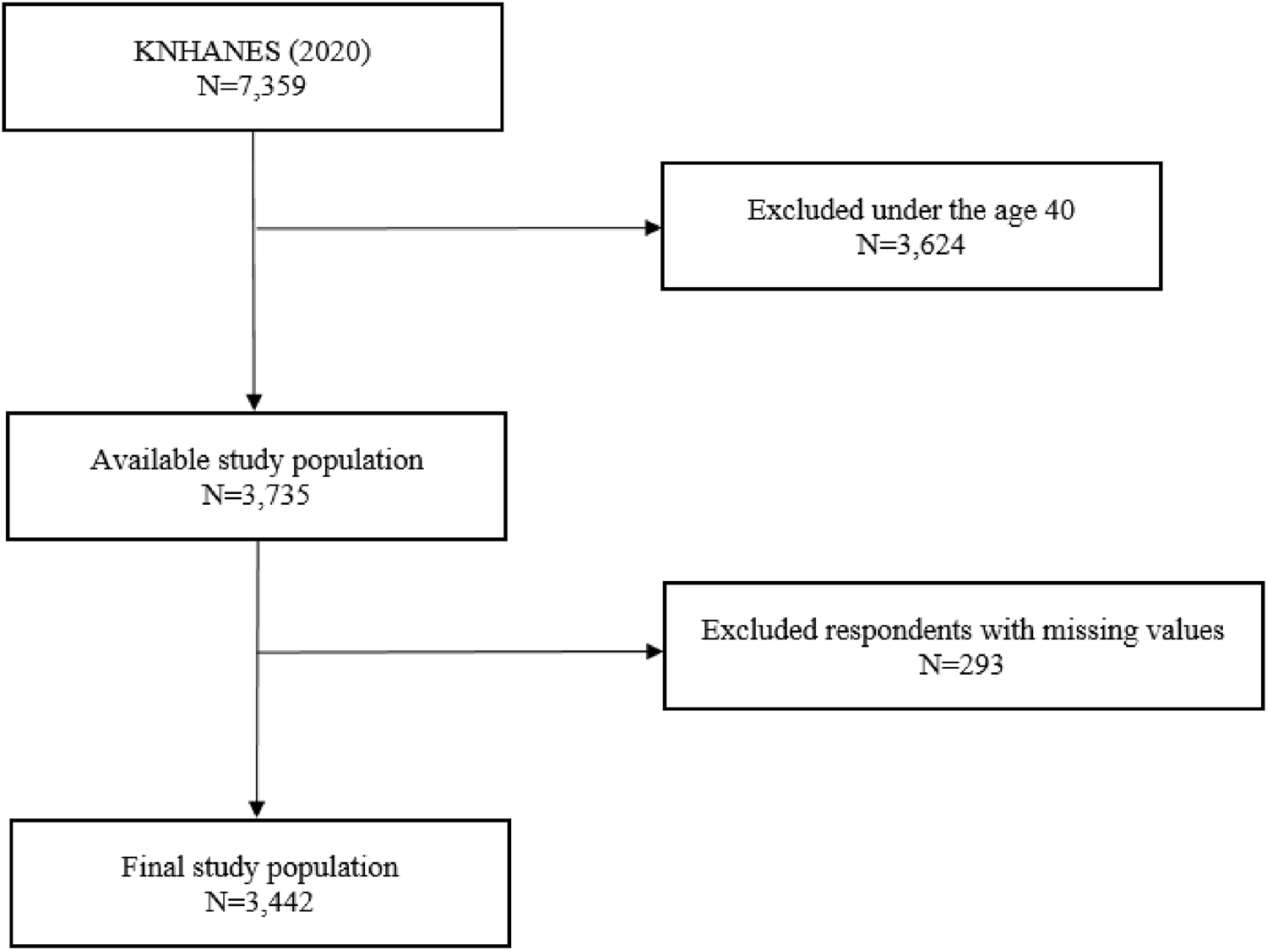
Flowchart of study participants displaying the inclusion and exclusion.

In 2020, the total number of respondents was 7359. Individuals aged between 1 and 18 years were excluded due to the lack of smoking information (*N* = 1,820). Additionally, respondents under age 40 were excluded because they were not measured using the STOP-Bang index (*N* = 1804). Participants with missing data were also excluded (*N* = 293). Finally, a sample of 3442 participants (1465 males and 1977 females) was analyzed in this study (Fig. 1).

### Variables

A dependent variable was defined by the STOP-Bang score, which reflects OSA risk using an 8-item questionnaire^7,30^. Polysomnography (PSG), a time and money-intensive complex procedure, is performed to diagnose OSA. Therefore, the STOP-Bang indicator is generally used^27,31^. The STOP-Bang questionnaire has high sensitivity and is a valid screening tool for OSA worldwide^32,33^. A relatively easy screening tool, STOP-Bang, has been validated with higher sensitivity than the Berlin questionnaire in South Korean patients^34^. KNHANES, which represents Koreans, introduced newly a STOP-Bang as a simple and user-friendly screening tool in 2019, and many research using it is being actively conducted. STOP-Bang is an acronym for the first letter of each symptom or physical attribute often associated with OSA, S, T, O, P, and BANG. The questionnaire included sleep-related symptoms, blood pressure, body mass index (BMI), neck circumference, age, and sex. Each option was rated as 0 (“No”) or 1(“Yes”), resulting in a maximum score of 8 points. In addition, sleep-related symptoms (snoring, tiredness, and observed apnea) were self-reported: (1) Is your snoring louder than the conversation or loud enough to be heard in the next room? (2) Do you often feel tired or sleepy during the day? and (3) Has anyone seen you stop breathing when you sleep? Blood pressure was measured using a non-mercury automatic sphygmomanometer (Microlife WatchBP Office AFIB), an internationally certified device. BMI was calculated as weight (kg) divided by height squared (m^2^). Neck circumference was measured using a Lufkin W606PM device below the thyroid cartilage (Adam’s apple). The risk of OSA was based on a global cutoff^32,33^, with a low risk of OSA if the score was ≤ 2 and a high risk if it was ≥ 3. In additional subgroup analysis, a cutoff was used by dividing the degree of OSA risk using the STOP-Bang score into three categories, with a mild risk if the score was from 0 to 2, moderate risk if the score was from 3 to 4, and severe risk if the score was from 5 to 8. Also, a high STOP-Bang score means a high risk of OSA, which does not confirm a full OSA diagnosis.

The main independent variable was “smoking,” which was classified into three groups: (1) non-smokers, (2) ex-smokers who had been using conventional or e-cigarettes previously, and (3) current smokers who use conventional or e-cigarettes. This classification was similar to that of previous studies investigating smoking behavior using the same tools^10^.

The covariates included demographic factors, socioeconomic factors, health conditions, and health behaviors of the participants. Demographic factors included age (40–49 years/50–59 years/60–69 years/ ≥ 70 years), marital status (married/single and widow/divorced and separated), and educational level (middle school or below/high school/college or over). Socioeconomic factors included household income (low/mid-low/mid-high/high), region (urban/rural), and occupational categories (white/pink/blue/inoccupation). Health conditions and health behaviors included high-risk drinking (no-drinker/low-risk drinker/high-risk drinker), physical activity (active/inactive), BMI (underweight and normal/overweight/obesity of stage 1/obesity of stage 2 and 3), status of hypertension (normal/warning/pre-/stage 1/stage 2), status of diabetes (normal/pre-/diabetes), allergic rhinitis history (yes/no), and life disturbance due to rhinitis (yes/no).

### Statistical analysis

All estimates were calculated using sample weight procedures, clusters, and strata assigned to the study participants. In addition, we analyzed each variable stratified by sex. Descriptive analysis was performed to examine the general characteristics of the study population. To assess the association between smoking and OSA (low risk/ high risk), we used multi-logistic regression analysis. Furthermore, a multinomial regression analysis stratified by dependent variables as OSA (mild/moderate/severe). Subgroup analyses stratified by interesting variable were performed to investigate the combined effects of OSA and indicators of severity of smoking through the smoking cessation status and pack-years among ex- and current smokers. In addition, we analyzed the association between moderate risk and severe risk compared to the mild risk of OSA, respectively (supplementary 1 and 2). The results were reported as odds ratios (ORs) and 95% confidence intervals (CIs). SAS version 9.4 (SAS Institute Inc.; Cary, NC, USA) was used for all the analyses, and a *p*-value of 0.05 was considered significant.

## Results

Table 1 presents the general characteristics of the study population. Of the 3442 participants, 1465 were males (42.6%) and 1977 were females (57.4%). Among the males, 435 (29.7%) were current smokers, 725 (49.5%) were ex-smokers, and 305 (20.8%) were non-smokers. Among the females, 61 (3.1%) were current smokers, 86 (4.4%) were ex-smokers, and 1830 (92.6%) were non-smokers. The relationship between smoking and OSA was significant in males (*p*-value = 0.0270).

**Table 1:** General characteristics of the study population.

Variables	Obstructive sleep apnea (OSA)													
	Men							Women						
	Total		High-risk		Low-risk		*p*-value	Total		High-risk		Low-risk		*p*-value
	N	%	N	%	N	%		N	%	N	%	N	%	
Total (*N* = 3442)	1465	100.0	535	36.5	930	63.5		1977	100.0	371	18.8	1606	81.2	
Smoking behavior							0.0270							0.6966
Non-smoker	305	20.8	183	60.0	122	40.0		1,830	92.6	341	18.6	1,489	81.4	
Ex-smoker	725	49.5	485	66.9	240	33.1		86	4.4	16	18.6	70	81.4	
Current smoker	435	29.7	262	60.2	173	39.8		61	3.1	14	23.0	47	77.0	
Age							< .0001							< .0001
40–49	306	20.9	99	32.4	207	67.6		482	24.4	13	2.7	469	97.3	
50–59	401	27.4	283	70.6	118	29.4		526	26.6	94	17.9	432	82.1	
60–69	388	26.5	272	70.1	116	29.9		549	27.8	143	26.0	406	74.0	
≥ 70	370	25.3	276	74.6	94	25.4		420	21.2	121	28.8	299	71.2	
Marital status							0.0212							0.1500
Married	1,238	84.5	797	64.4	441	35.6		1,457	73.7	262	18.0	1,195	82.0	
Single, widow	128	8.7	67	52.3	61	47.7		362	18.3	81	22.4	281	77.6	
Divorced, separated	99	6.8	66	66.7	33	33.3		158	8.0	28	17.7	130	82.3	
Educational level							< .0001							< .0001
Middle school or below	432	29.5	323	74.8	109	25.2		804	40.7	217	27.0	587	73.0	
High school	481	32.8	301	62.6	180	37.4		640	32.4	98	15.3	542	84.7	
College or over	552	37.7	306	55.4	246	44.6		533	27.0	56	10.5	477	89.5	
Household income							0.0049							< .0001
Low	265	18.1	189	71.3	76	28.7		426	21.5	116	27.2	310	72.8	
Mid-low	355	24.2	235	66.2	120	33.8		493	24.9	97	19.7	396	80.3	
Mid-high	403	27.5	242	60.0	161	40.0		533	27.0	91	17.1	442	82.9	
High	442	30.2	264	59.7	178	40.3		525	26.6	67	12.8	458	87.2	
Region							0.0302							0.0179
Urban	1,123	76.7	696	62.0	427	38.0		1,570	79.4	278	17.7	1,292	82.3	
Rural	342	23.3	234	68.4	108	31.6		407	20.6	93	22.9	314	77.1	
Occupational categories							< .0001							< .0001
White	378	25.8	204	54.0	174	46.0		323	16.3	27	8.4	296	91.6	
Pink	126	8.6	72	57.1	54	42.9		320	16.2	53	16.6	267	83.4	
Blue	521	35.6	341	65.5	180	34.5		344	17.4	65	18.9	279	81.1	
Inoccupation	440	30.0	313	71.1	127	28.9		990	50.1	226	22.8	764	77.2	
High-risk drinking							0.0328							0.0029
No-drinker	73	5.0	48	65.8	25	34.2		410	20.7	101	24.6	309	75.4	
Low-risk drinker	1,112	75.9	686	61.7	426	38.3		1,506	76.2	260	17.3	1,246	82.7	
High-risk drinker	280	19.1	196	70.0	84	30.0		61	3.1	10	16.4	51	83.6	
Physical activity							0.7379							0.0523
Active	849	58.0	542	63.8	307	36.2		726	36.7	251	34.6	606	83.5	
Inactive	616	42.0	388	63.0	228	37.0		1,251	63.3	120	9.6	1,000	79.9	
BMI							< .0001							< .0001
Underweight and normal	407	27.8	216	53.1	191	46.9		853	43.1	91	10.7	762	89.3	
Overweight	400	27.3	249	62.3	151	37.8		439	22.2	77	17.5	362	82.5	
Obesity of stage 1	579	39.5	398	68.7	181	31.3		573	29.0	161	28.1	412	71.9	
Obesity of stage 2&3	79	5.4	67	84.8	12	15.2		112	5.7	42	37.5	70	62.5	
Status of hypertension							< .0001							< .0001
Normal	352	24.0	115	32.7	237	67.3		767	38.8	36	4.7	731	95.3	
Warning	101	6.9	41	40.6	60	59.4		139	7.0	11	7.9	128	92.1	
Pre-hypertension	335	22.9	136	40.6	199	59.4		338	17.1	22	6.5	316	93.5	
Hypertension of stage 1	150	10.2	135	90.0	15	10.0		144	7.3	50	34.7	94	65.3	
Hypertension of stage 2	527	36.0	503	95.4	24	4.6		589	29.8	252	42.8	337	57.2	
Status of diabetes							< .0001							< .0001
Normal	397	27.1	212	53.4	185	46.6		695	35.2	70	10.1	625	89.9	
Pre-diabetes	716	48.9	465	64.9	251	35.1		926	46.8	185	20.0	741	80.0	
Diabetes	352	24.0	253	71.9	99	28.1		356	18.0	116	32.6	240	67.4	
Allergic rhinitis history							0.7395							0.7652
Yes	153	10.4	99	64.7	54	35.3		294	14.9	57	19.4	237	80.6	
No	1312	89.6	831	63.3	481	36.7		1683	85.1	314	18.7	1,369	81.3	
Life disturbance due to Rhinitis							0.0297							0.5660
Yes	142	9.7	102	71.8	40	28.2		223	11.3	45	20.2	178	79.8	
No	1,323	90.3	828	62.6	495	37.4		1,754	88.7	326	18.6	1,428	81.4	

Table 2 presents the association between smoking and OSA in males and females after adjusting for all covariates. Among the males, the ex-smokers (OR: 1.53, 95% CI 1.01–2.32) and current smokers (OR: 1.79, 95% CI 1.10–2.89) had a significantly higher association with OSA than the non-smokers. Among the females, the ex-smokers and current smokers exhibited an increasing trend of ORs for a high risk of OSA, although the association with OSA was not significant.

**Table 2:** Results of factors associated between smoking and obstructive sleep apnea.

Variables	Obstructive sleep apnea (OSA)					
	Men		Women			
	OR	95% CI	OR	95% CI		
Smoking behavior						
Non–smoker	1.00		1.00			
Ex–smoker	1.53*	(1.01–2.32)	1.62	(0.66–3.97)		
Current smoker	1.79*	(1.10–2.89)	1.66	(0.60–4.57)		
Age						
40–49	1.00		1.00			
50–59	8.34*	(4.63–15.03)	4.72*	(2.38–9.37)		
60–69	6.97*	(4.01–12.11)	5.27*	(2.70–10.29)		
≥ 70	6.50*	(3.56–11.88)	4.16*	(1.80–9.63)		
Marital status						
Married	1.00		1.00			
Single, widow	0.68	(0.34–1.34)	0.65	(0.42–0.99)		
Divorced, separated	0.64	(0.35–1.15)	0.65	(0.37–1.13)		
Educational level						
Middle school or below	1.00		1.00			
High school	0.74	(0.45–1.23)	1.20	(0.78–1.84)		
College or over	0.88	(0.49–1.59)	1.14	(0.64–2.00)		
Household income						
Low	1.00		1.00			
Mid–low	1.24	(0.70–2.17)	0.80	(0.50–1.27)		
Mid–high	1.24	(0.72–2.11)	0.76	(0.51–1.13)		
High	1.27	(0.69–2.35)	0.70	(0.42–1.19)		
Region						
Urban	1.00		1.00			
Rural	1.19	(0.74–1.92)	1.13	(0.74–1.73)		
Occupational categories						
White	1.12	(0.64–1.97)	0.77	(0.38–1.59)		
Pink	0.77	(0.40–1.48)	1.07	(0.63–1.80)		
Blue	0.84	(0.55–1.29)	0.79	(0.52–1.20)		
Inoccupation	1.00		1.00			
High–risk drinking						
No–drinker	1.00		1.00			
Low–risk drinker	0.90	(0.39–2.07)	0.95	(0.64–1.42)		
High–risk drinker	1.15	(0.47–2.84)	0.77	(0.34–1.73)		
Physical activity						
Active	1.00		1.00			
Inactive	1.05	(0.71–1.55)	1.12	(0.79–1.58)		
BMI						
Underweight and normal	1.00		1.00			
Overweight	1.19	(0.80–1.75)	1.55	(0.96–2.52)		
Obesity of stage 1	1.41	(0.93–2.15)	2.27*	(1.52–3.40)		
Obesity of stage 2&3	3.44*	(1.17–10.09)	3.33*	(1.84–6.03)		
Status of hypertension						
Normal	1.00		1.00			
Warning	1.20	(0.67–2.16)	1.26	(0.57–2.78)		
Pre–hypertension	1.38	(0.85–2.23)	1.31	(0.64–2.70)		
Hypertension of stage 1	18.92*	(8.61–41.58)	10.51*	(5.98–18.47)		
Hypertension of stage 2	29.38*	(16.65–51.85)	10.83*	(6.12–19.17)		
Status of diabetes						
Normal	1.00		1.00			
Pre–diabetes	1.10	(0.72–1.68)	0.88	(0.60–1.30)		
Diabetes	1.02	(0.60–1.68)	1.18	(0.72–1.93)		
Allergic rhinitis history						
Yes	1.44	(0.76–2.73)	1.72	(0.95–3.10)		
No	1.00		1.00			
Life disturbance due to Rhinitis						
Yes	1.89*	(1.03–3.47)	0.88	(0.49–1.56		
No	1.00		1.00			

*Statistically significant in men or women.

Table 3 presents the results of a multinomial regression analysis of the three groups in the STOP-Bang index based on sex. It shows the results of a stratified analysis of the association between smoking and the degree of OSA risk. The degree of OSA risk was based on a global cutoff using the STOP-Bang score^32,33^: mild risk (0–2), moderate risk (3–4), and severe risk (5–8). Considering the mild risk of OSA as the reference category, the ORs of smoking status were linearly higher in ex-smokers and current smokers. In males, ex-smokers (OR: 1.61, 95% CI 1.05–2.48) were significantly associated with a moderate risk of OSA, and current smokers (OR: 1.88, 95% CI 1.07–3.29) were significantly associated with a severe risk of OSA.

**Table 3:** Multinomial regression of three-groups in STOP-BANG index.

Variables†		Obstructive sleep Apnea (Ref: mild)			
		Moderate		Severe	
		OR	9% CI	OR	95% CI
Men	Non-smoker	1.00		1.00	
	Ex-smoker	1.61	(1.05–2.48)	1.51	(0.90–2.55)
	Current smoker	1.68	(0.98–2.86)	1.88	(1.07–3.29)
Women	Non-smoker	1.00		1.00	
	Ex-smoker	1.64	(0.67–3.99)	2.00	(0.17–23.50)
	Current smoker	1.85	(0.69–4.95)	*	*

^†^Adjusted for all covariates.

*Due to sparsity of the data, OR could not be calculated in the model.

**Figure 2:**
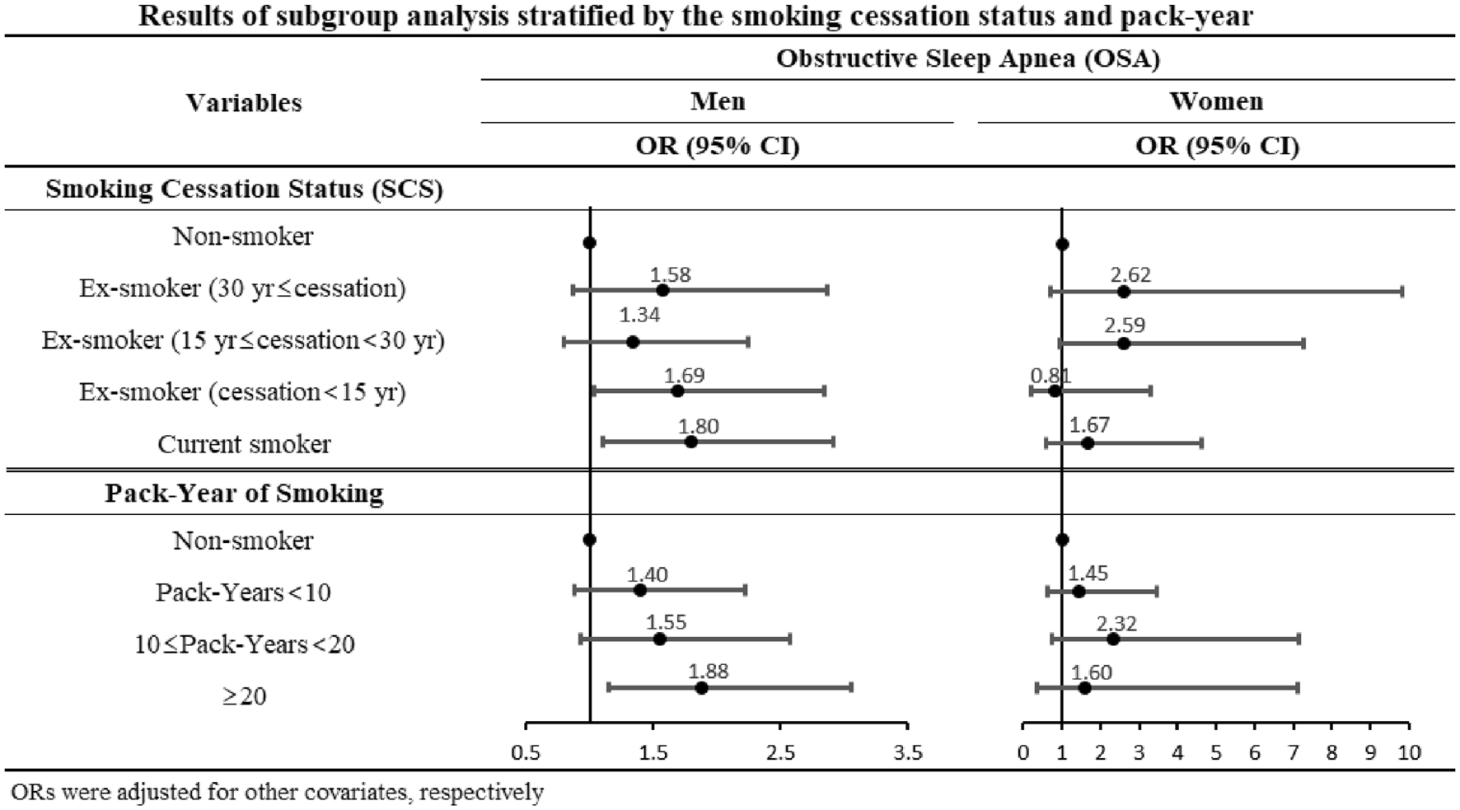
Results of subgroup analysis stratified by the smoking cessation status and pack-year.

Figure 2 presents the results of the subgroup analysis of changes in ORs based on smoking cessation status (SCS) and pack-years (the number of cigarettes smoked and the smoking period). The ORs increased linearly as the SCS and pack-years increased in males. Specifically, ex-smokers with < 15 years of smoking cessation (OR: 1.69, 95% CI 1.01–2.84), current smokers (OR: 1.80, 95% CI 1.11–2.91), and over 20 pack-years (OR: 1.88, 95% CI 1.15–3.05) were more likely to have high ORs for OSA compared to non-smokers.

## Discussion

In this study, we observed that OSA risk was higher in ex-smokers and current smokers than in non-smokers in males. In addition, the risk had a positive dose-dependent relationship with pack-years and negative with the duration of smoking cessation. According to the STOP-Bang, there was a significant association between a higher OSA risk and ex-smokers and the highest OSA risk and current smokers. In other words, current smokers quitting will contribute to a reduced OSA risk. However, we did not observe a significant association between smoking and OSA due to the few female smokers. Nevertheless, the increasing ORs based on smoking behavior were similar in men. This can be attributed to the low awareness of smoking among Korean women, regarded as a recall bias in self-reported data^35–38^. This underreporting of women’s smoking is also linked to the critical view of society. That’s because the social stigma of women smokers tends to hide and cover up smoking more than men^39^.

According to previous studies, the association between smoking and OSA has many conflicting opinions, although many research groups have not studied it. A study observed that patients with OSA had more current smokers than those without OSA^40^. This finding suggests that smoking may be an independent risk factor for OSA^40^. Also, it is an independent risk factor for intermittent hypoxemia, and smokers with OSA have been associated with the most severe endothelial function impairment^41^. It occurs more frequently in children exposed to secondary smoking, and adults smokers^42,43^, and these children exhibit more concentration challenges, fatigue, irritability, and hyperactivity due to OSA^42^. Conversely, a study analyzed medical records and polysomnography results and observed no significant correlation between smoking and OSA^44–46^. Additionally, a relationship between smoking and OSA is plausible; however, there is no conclusive evidence suggesting that untreated OSA is associated with smoking addiction^26^. However, categorizing the medical records and polysomnography results revealed that smokers with a low BMI developed OSA, and heavy smokers had a light sleep stage and a high OSA risk^44^. The most likely explanation for results that differ from this study is the measurement methods of OSA and study population and year.

Several mechanisms have been proposed to explain the association between smoking and OSA. First, we described some biological mechanisms. Smoking alters the uveal mucosa in patients with OSA, resulting in thickening and edema through calcitonin gene-related peptide (CGRP)-induced neurogenic inflammation^47^. Nasal obstruction due to chronic mucosal inflammation associated with smoking, such as impaired ciliary function, mucosal edema, cell proliferation, and thickened epithelium, is also considered a potential mechanism^48–50^. This supports our findings that prolonged smoking increases the risk of moderate or severe OSA. Mild risk of OSA are common, and typically untreated^51^. Moderate and severe risk of OSA have been well established to increase risk in the presence of incident cardiometabolic comorbidities such as heart failure, ischemic heart disease, hypertension, diabetes, and so on^51^. If people with moderate or severe risk of OSA are not treated, the risk of death from cardiovascular and all causes increases significantly^52–54^, so they should always be prevented and monitored.

Second, other potential associations between smoking and OSA have been suggested as nicotine-induced impairment from neuromuscular protection reflexes in the upper airway^55^. Finally, smoking can lead to OSA due to its respiratory effects, nicotine withdrawal, and the stimulant effects of nicotine. According to previous studies, smokers are more likely to experience daytime sleepiness^56,57^ and respiratory sleep disorders than non-smokers^58^.

On the other hand, Gothe et al.^59^ demonstrated that chewing nicotine gum before sleep reduces the frequency of obstructive apnea episodes during the first two hours of sleep. This suggests that nicotine can reduce sleep breathing disorders^59^. However, during the night, as nicotine blood levels decreases and upper airway resistance increases, the apnea–hypopnea index (AHI) increases with nicotine withdrawal symptoms or respiratory effects associated with smoking^59^.

This study had certain limitations. First, it was a cross-sectional study. It may be an inverse causal relationship; therefore, caution should be exercised in the interpretation. Future studies are required to clarify the relationship between smoking and OSA, and longitudinal studies are required to establish a causal relationship. Second, KNHANES data were self-reported. Data on smoking status, the STOP-Bang index, and health-related and socioeconomic variables might be reliable but not accurately measured. In addition, the STOP-Bang index indicates OSA risk, not its prevalence. It may result in recall bias, which has been underestimated in smoking. However, we minimized this bias by using a globally reliable STOP-Bang index. Third, the available results regarding the causes of the STOP-Bang index were only available for 2020, and the KNHANES data for other years could not be obtained. If STOP-Bang data continue to emerge in future KNHANES, studies using a larger study population would be required. Fourth, STOP-Bang index has not been investigated for people under the age of 40, so our participants are adults over the age of 40. This is the limitation of the data, and future studies need to be conducted for all age groups. The results of this study cannot be generalized to young people at OSA risk. Fifth, the STOP-Bang index is not an objective measurement method to define OSA. However, it is mostly used as a screening tool in place of polysomnography. Therefore, further research using the test result data for polysomnography is needed. Finally, we could not identify the type and smoking behaviors, such as conventional cigarette smoking, e-cigarette smoking, or both. Furthermore, the pack-years of liquid e-cigarettes could not be calculated because the KNHANES did not contain this information.

Despite these limitations, our study had notable strengths. First, this study was based on the KNHANES, a nationally representative data collected by the KDCA. It is conducted using random cluster sampling, which is reliable and representative. Therefore, the study results can be generalized to adults over the age of 40 in Korea. Second, to the best of our knowledge, this is the first study to investigate the association between smoking and OSA using the STOP-Bang index, an appropriate tool for measuring OSA in the general population. Our research suggests appropriate interventions to improve sleep quality in smokers at risk of OSA based on the STOP-Bang index should be developed and applied.

## Conclusion

In conclusion, our study suggests that smoking may be associated with OSA and may further affect sleep quality. Given these results, current smokers are at risk of OSA; however, it is also related to smoking cessation and pack-years, which are calculated by the amount and duration of smoking. These findings provide direction for future studies on smoking disadvantages and OSA and educate patients with OSA who are unaware of it. Knowing our health status and quitting smoking are the best ways to avoid life-threatening diseases.

## Supplementary Information

Supplementary Table S1.

Supplementary Table S2.

Supplementary Figure S1.

## Data Availability

The datasets generated and/or analyzed during the current study are available in the Korea National Health and Nutrition Examination Survey (KNHANES) 2020, https://kdca.go.kr/index.es?sid=a2.
