# Supplementary material for: Association between smoking and obstructive sleep apnea based on the STOP-Bang index
Source: Sci Rep. 2023 Jun 5;13:9085. doi: 10.1038/s41598-023-34956-5 (PMC10241803; doi:10.1038/s41598-023-34956-5)
Supplement: Supplementary file 1 — Supplementary Table S1. [file 41598_2023_34956_MOESM1_ESM.docx]

| **Supplementary 1. Results of factors associated between smoking and obstructive sleep apnea except for the severe risk of OSA (ref='mild')** | | | | | | | | | | |
| --- | --- | --- | --- | --- | --- | --- | --- | --- | --- | --- |
| **Variables** | | **Moderate risk of Obstructive Sleep Apnea (OSA)†** | | | | | | | | |
|  |  | **Men** | | | |  | **Women** | | | |
|  |  | **OR** | **95% CI** | | |  | **OR** | **95% CI** | | |
| **Smoking Behavior** | |  |  |  |  |  |  |  |  |  |
|  | Non-smoker | 1.00 |  |  |  |  | 1.00 |  |  |  |
|  | Ex-smoker | 1.52 | (0.91 | - | 2.52) |  | 1.67 | (0.70 | - | 4.00) |
|  | Current smoker | 1.76 | (0.92 | - | 3.36) |  | 1.85 | (0.70 | - | 4.91) |
| **Age** |  |  |  |  |  |  |  |  |  |  |
|  | 40-49 | 1.00 |  |  |  |  | 1.00 |  |  |  |
|  | 50-59 | * | * | * | * |  | 5.44 | (2.61 | - | 11.34) |
|  | 60-69 | * | * | * | * |  | 6.20 | (2.97 | - | 12.97) |
|  | ≥70 | * | * | * | * |  | 5.25 | (2.25 | - | 12.27) |
| **Marital status** | |  |  |  |  |  |  |  |  |  |
|  | Married | 1.00 |  |  |  |  | 1.00 |  |  |  |
|  | Single, widow | 0.76 | (0.31 | - | 1.85) |  | 0.64 | (0.42 | - | 0.99) |
|  | Divorced, Separated | 0.51 | (0.21 | - | 1.26) |  | 0.62 | (0.34 | - | 1.12) |
| **Educational level** | |  |  |  |  |  |  |  |  |  |
|  | Middle school or below | 1.00 |  |  |  |  | 1.00 |  |  |  |
|  | High school | 0.46 | (0.25 | - | 0.85) |  | 1.20 | (0.76 | - | 1.89) |
|  | College or over | 0.52 | (0.26 | - | 1.06) |  | 1.10 | (0.61 | - | 1.97) |
| **Household income** | |  |  |  |  |  |  |  |  |  |
|  | Low | 1.00 |  |  |  |  | 1.00 |  |  |  |
|  | Mid-low | 1.24 | (0.60 | - | 2.56) |  | 0.87 | (0.54 | - | 1.41) |
|  | Mid-high | 1.61 | (0.85 | - | 3.04) |  | 0.81 | (0.54 | - | 1.23) |
|  | High | 1.59 | (0.82 | - | 3.12) |  | 0.71 | (0.42 | - | 1.20) |
| **Region** | |  |  |  |  |  |  |  |  |  |
|  | Urban | 1.00 |  |  |  |  | 1.00 |  |  |  |
|  | Rural | 1.12 | (0.65 | - | 1.92) |  | 1.21 | (0.79 | - | 1.86) |
| **Occupational categories** | |  |  |  |  |  |  |  |  |  |
|  | White | 0.81 | (0.41 | - | 1.58) |  | 0.82 | (0.39 | - | 1.73) |
|  | Pink | 0.59 | (0.27 | - | 1.31) |  | 1.03 | (0.59 | - | 1.80) |
|  | Blue | 0.70 | (0.41 | - | 1.20) |  | 0.81 | (0.53 | - | 1.23) |
|  | Inoccupation | 1.00 |  |  |  |  | 1.00 |  |  |  |
| **High-risk drinking** | |  |  |  |  |  |  |  |  |  |
|  | No-drinker | 1.00 |  |  |  |  | 1.00 |  |  |  |
|  | Low-risk drinker | 0.89 | (0.42 | - | 1.92) |  | 1.09 | (0.72 | - | 1.65) |
|  | High-risk drinker | 1.49 | (0.59 | - | 3.78) |  | 0.87 | (0.37 | - | 2.07) |
| **Physical activity** | |  |  |  |  |  |  |  |  |  |
|  | Active | 1.00 |  |  |  |  | 1.00 |  |  |  |
|  | Inactive | 0.81 | (0.48 | - | 1.39) |  | 1.06 | (0.74 | - | 1.52) |
| **BMI** |  |  |  |  |  |  |  |  |  |  |
|  | Underweight and normal | 1.00 |  |  |  |  | 1.00 |  |  |  |
|  | Overweight | 0.91 | (0.57 | - | 1.44) |  | 1.63 | (0.99 | - | 2.67) |
|  | Obesity of stage 1 | 1.10 | (0.62 | - | 1.95) |  | 2.26 | (1.49 | - | 3.43) |
|  | Obesity of stage 2&3 | 7.46 | (1.17 | - | 47.48) |  | 2.64 | (1.39 | - | 5.02) |
| **Status of hypertension** | |  |  |  |  |  |  |  |  |  |
|  | Normal | 1.00 |  |  |  |  | 1.00 |  |  |  |
|  | Warning | 1.86 | (0.95 | - | 3.65) |  | 1.11 | (0.50 | - | 2.43) |
|  | Pre-hypertension | 1.23 | (0.67 | - | 2.28) |  | 1.32 | (0.64 | - | 2.72) |
|  | Hypertension of stage 1 | 21.47 | (6.62 | - | 69.68) |  | 9.78 | (5.45 | - | 17.56) |
|  | Hypertension of stage 2 | 83.21 | (31.64 | - | 218.80) |  | 9.69 | (5.46 | - | 17.21) |
| **Status of diabetes** | |  |  |  |  |  |  |  |  |  |
|  | Normal | 1.00 |  |  |  |  | 1.00 |  |  |  |
|  | Pre-diabetes | 1.44 | (0.90 | - | 2.30) |  | 0.89 | (0.60 | - | 1.33) |
|  | Diabetes | 1.09 | (0.56 | - | 2.14) |  | 1.20 | (0.72 | - | 2.00) |
| **Allergic rhinitis history** | |  |  |  |  |  |  |  |  |  |
|  | Yes | 1.24 | (0.52 | - | 2.96) |  | 1.80 | (0.99 | - | 3.26) |
|  | No | 1.00 |  |  |  |  | 1.00 |  |  |  |
| **Life disturbance due to Rhinitis** | |  |  |  |  |  |  |  |  |  |
|  | Yes | 1.41 | (0.69 | - | 2.89) |  | 0.82 | (0.46 | - | 1.44) |
|  | No | 1.00 |  |  |  |  | 1.00 |  |  |  |
| † The analysis was performed including participants who has the risk of mild and moderate of OSA. * Due to sparsity of the data, OR could not be calculated in the model | | | | | | | | | | |
