# Supplementary material for: Association between smoking and obstructive sleep apnea based on the STOP-Bang index
Source: Sci Rep. 2023 Jun 5;13:9085. doi: 10.1038/s41598-023-34956-5 (PMC10241803; doi:10.1038/s41598-023-34956-5)
Supplement: Supplementary file 2 — Supplementary Table S2. [file 41598_2023_34956_MOESM2_ESM.docx]

| **Supplementary 2. Results of factors associated between smoking and obstructive sleep apnea except for the moderate risk of OSA (ref='mild')** | | | | | | | | | | |
| --- | --- | --- | --- | --- | --- | --- | --- | --- | --- | --- |
| **Variables** | | **Severe risk of Obstructive Sleep Apnea (OSA)†** | | | | | | | | |
|  |  | **Men** | | | |  | **Women** | | | |
|  |  | **OR** | **95% CI** | | |  | **OR** | **95% CI** | | |
| **Smoking Behavior** | |  |  |  |  |  |  |  |  |  |
|  | Non-smoker | 1.00 |  |  |  |  | 1.00 |  |  |  |
|  | Ex-smoker | 1.53 | (0.88 | - | 2.69) |  | 0.56 | (0.03 | - | 12.16) |
|  | Current smoker | 1.95 | (1.05 | - | 3.60) |  | * | * | * | * |
| **Age** |  |  |  |  |  |  |  |  |  |  |
|  | 40-49 | 1.00 |  |  |  |  | 1.00 |  |  |  |
|  | 50-59 | 4.39 | (2.31 | - | 8.34) |  | 1.15 | (0.18 | - | 7.13) |
|  | 60-69 | 4.09 | (2.05 | - | 8.18) |  | 1.50 | (0.28 | - | 8.06) |
|  | ≥70 | 4.38 | (2.20 | - | 8.72) |  | 0.36 | (0.05 | - | 2.50) |
| **Marital status** | |  |  |  |  |  |  |  |  |  |
|  | Married | 1.00 |  |  |  |  | 1.00 |  |  |  |
|  | Single, widow | 0.69 | (0.30 | - | 1.61) |  | 0.45 | (0.10 | - | 1.92) |
|  | Divorced, Separated | 0.71 | (0.35 | - | 1.45) |  | 1.22 | (0.14 | - | 10.92) |
| **Educational level** | |  |  |  |  |  |  |  |  |  |
|  | Middle school or below | 1.00 |  |  |  |  | 1.00 |  |  |  |
|  | High school | 1.40 | (0.78 | - | 2.49) |  | 3.00 | (0.42 | - | 21.61) |
|  | College or over | 1.83 | (0.94 | - | 3.56) |  | 0.86 | (0.14 | - | 5.25) |
| **Household income** | |  |  |  |  |  |  |  |  |  |
|  | Low | 1.00 |  |  |  |  | 1.00 |  |  |  |
|  | Mid-low | 1.09 | (0.57 | - | 2.11) |  | 0.14 | (0.01 | - | 1.53) |
|  | Mid-high | 0.94 | (0.50 | - | 1.79) |  | 0.15 | (0.02 | - | 1.34) |
|  | High | 0.98 | (0.47 | - | 2.03) |  | 0.86 | (0.09 | - | 8.27) |
| **Region** |  |  |  |  |  |  |  |  |  |  |
|  | Urban | 1.00 |  |  |  |  | 1.00 |  |  |  |
|  | Rural | 1.28 | (0.69 | - | 2.38) |  | 0.12 | (0.01 | - | 1.27) |
| **Occupational categories** | |  |  |  |  |  |  |  |  |  |
|  | White | 1.55 | (0.75 | - | 3.23) |  | 0.81 | (0.11 | - | 6.21) |
|  | Pink | 1.16 | (0.53 | - | 2.55) |  | 2.30 | (0.51 | - | 10.40) |
|  | Blue | 1.16 | (0.62 | - | 2.16) |  | 0.85 | (0.15 | - | 4.70) |
|  | Inoccupation | 1.00 |  |  |  |  | 1.00 |  |  |  |
| **High-risk drinking** | |  |  |  |  |  |  |  |  |  |
|  | No-drinker | 1.00 |  |  |  |  | 1.00 |  |  |  |
|  | Low-risk drinker | 0.84 | (0.26 | - | 2.70) |  | 0.11 | (0.02 | - | 0.47) |
|  | High-risk drinker | 0.98 | (0.29 | - | 3.33) |  | 0.51 | (0.05 | - | 5.62) |
| **Physical activity** | |  |  |  |  |  |  |  |  |  |
|  | Active | 1.00 |  |  |  |  | 1.00 |  |  |  |
|  | Inactive | 1.20 | (0.74 | - | 1.93) |  | 4.56 | (1.52 | - | 13.70) |
| **BMI** |  |  |  |  |  |  |  |  |  |  |
|  | Underweight and normal | 1.00 |  |  |  |  | 1.00 |  |  |  |
|  | Overweight | 1.47 | (0.88 | - | 2.45) |  | 0.06 | (0.01 | - | 0.69) |
|  | Obesity of stage 1 | 1.74 | (1.10 | - | 2.76) |  | 1.79 | (0.45 | - | 7.12) |
|  | Obesity of stage 2&3 | 3.29 | (0.96 | - | 11.21) |  | 26.82 | (4.99 | - | 144.13) |
| **Status of hypertension** | |  |  |  |  |  |  |  |  |  |
|  | Normal | 1.00 |  |  |  |  | 1.00 |  |  |  |
|  | Warning | 0.56 | (0.22 | - | 1.42) |  | * | * | * | * |
|  | Pre-hypertension | 1.61 | (0.89 | - | 2.92) |  | * | * | * | * |
|  | Hypertension of stage 1 | 23.02 | (10.57 | - | 50.17) |  | * | * | * | * |
|  | Hypertension of stage 2 | 32.39 | (16.01 | - | 65.53) |  | * | * | * | * |
| **Status of diabetes** | |  |  |  |  |  |  |  |  |  |
|  | Normal | 1.00 |  |  |  |  | 1.00 |  |  |  |
|  | Pre-diabetes | 1.03 | (0.62 | - | 1.72) |  | 1.03 | (0.26 | - | 4.00) |
|  | Diabetes | 1.11 | (0.56 | - | 2.19) |  | 1.06 | (0.21 | - | 5.38) |
| **Allergic rhinitis history** | |  |  |  |  |  |  |  |  |  |
|  | Yes | 1.48 | (0.70 | - | 3.14) |  | 1.40 | (0.24 | - | 8.25) |
|  | No | 1.00 |  |  |  |  | 1.00 |  |  |  |
| **Life disturbance due to Rhinitis** | |  |  |  |  |  |  |  |  |  |
|  | Yes | 2.38 | (1.16 | - | 4.87) |  | 1.36 | (0.25 | - | 7.40) |
|  | No | 1.00 |  |  |  |  | 1.00 |  |  |  |
| † The analysis was performed including participants who has the risk of mild and severe of OSA. * Due to sparsity of the data, OR could not be calculated in the model | | | | | | | | | | |
