# Supplementary material for: Association between smoking and obstructive sleep apnea based on the STOP-Bang index
Source: Sci Rep. 2023 Jun 5;13:9085. doi: 10.1038/s41598-023-34956-5 (PMC10241803; doi:10.1038/s41598-023-34956-5)
Supplement: Supplementary file 3 — Supplementary Figure S1. [file 41598_2023_34956_MOESM3_ESM.pdf]

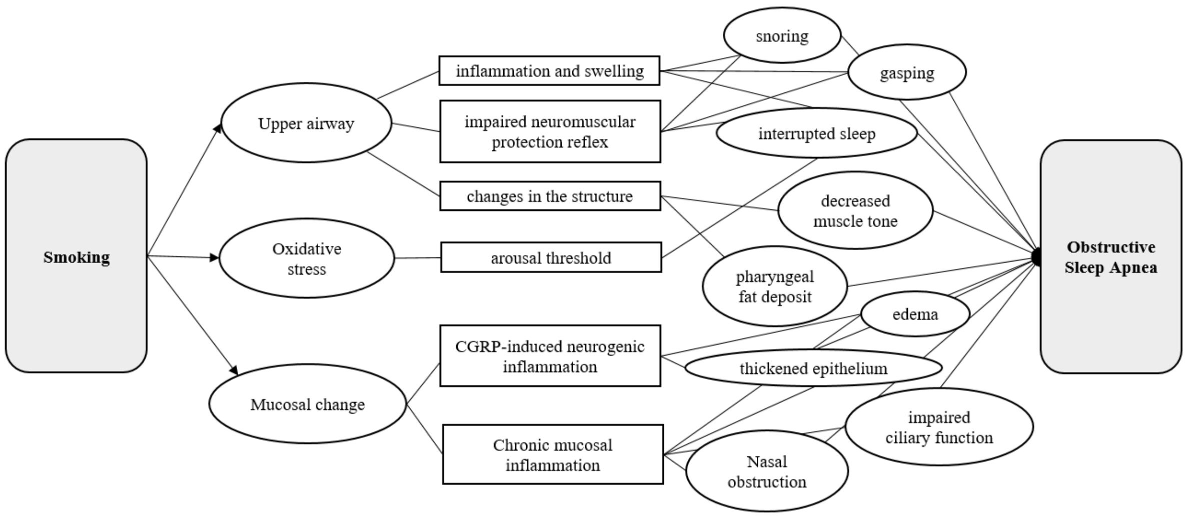


**Supplementary 3. Conceptual framework of the study, which is summary of the association between smoking and obstructive sleep apnea**
